# A comparative study between methylprednisolone versus dexamethasone as an initial anti-inflammatory treatment of moderate COVID-19 pneumonia: an open-label randomized controlled trial

**DOI:** 10.1186/s12890-024-03364-4

**Published:** 2024-11-11

**Authors:** Jakkrit Laikitmongkhon, Tanapat Tassaneyasin, Yuda Sutherasan, Angsana Phuphuakrat, Sirawat Srichatrapimuk, Tananchai Petnak, Dararat Eksombatchai, Kanin Thammavaranucupt, Somnuek Sungkanuparph

**Affiliations:** 1 Division of Pulmonary and Pulmonary Critical Care Medicine, Department of Medicine, Faculty of Medicine Ramathibodi Hospital, Mahidol University, 270 Rama VI Road, Ratchathewi, Bangkok, 10400 Thailand; 2 Faculty of Medicine Ramathibodi Hospital, Chakri Naruebodindra Medical Institute, Mahidol University, Samut Prakan, Thailand; 3 Division of Infectious Diseases, Department of Medicine, Faculty of Medicine Ramathibodi Hospital, Mahidol University, Bangkok, Thailand

**Keywords:** COVID-19, Moderate pneumonia, Corticosteroids, Randomized controlled trial

## Abstract

**Background:**

The most appropriate anti-inflammatory treatment for moderate COVID-19 pneumonia remains uncertain. We aimed to compare the effectiveness of a high-dose methylprednisolone versus a high-dose dexamethasone in hospitalized moderate COVID-19 pneumonia, regarding the WHO clinical progression scales, mortality, and the length of hospitalization.

**Methods:**

In this open-labeled randomized controlled trial, we enrolled patients with age > 18 years old who were diagnosed moderate COVID-19 pneumonia confirmed by real-time PCR, evidence of pneumonia by chest imaging and resting oxygen saturation between 90 and 94%. Patients were randomized at a 1:1 ratio to receive methylprednisolone 250 mg/day or dexamethasone 20 mg/day over the first three days. Then the patients in both groups received dexamethasone 20 mg/day on days 4–5, and 10 mg/day on days 6–10. Primary outcome was assessed by a 10-point WHO clinical progression scales ranging from uninfected (point 0) to death (point 10) on the fifth day of treatment. Secondary outcomes including 90-day mortality, length of hospitalization, rate of intensive care unit (ICU) transfer and complications were determined.

**Results:**

Of 98 eligible patients, the mean age was 76.0 ± 13.3 years. The median date of illness at the time of randomization was 3 days (interquartile range 2, 5). Baseline clinical characteristics and severity did not differ between groups. The WHO clinical progression scales were similar between methylprednisolone and dexamethasone group at 5 and 10 days of treatment [4.84, (95% confidence interval(CI), 4.35–5.33) vs. 4.76 (95% CI, 4.27–5.25), *p* = 0.821 and 4.32 (95% CI, 3.83–4.81) vs. 3.80 (95% CI, 3.31–4.29), *p* = 0.140, respectively)]. Both groups did not differ in-hospital mortality, length of hospitalization, and rate of ICU transfer. There were also no differences in steroid-related complications between groups until 90 days of follow-up.

**Conclusions:**

In patients with moderate COVID-19 pneumonia, initial anti-inflammatory treatment with 250 mg/day of methylprednisolone for three days does not yield better outcomes over high-dose dexamethasone.

**Trial registration:**

This study was registered at Thai Clinical Trials Registry on October 17, 2021, with the identifier TCTR20211017001.

**Supplementary Information:**

The online version contains supplementary material available at 10.1186/s12890-024-03364-4.

## Background

During the outbreak of the COVID-19, the public health system is overburdened with cases. Severe and critical cases have a higher mortality rate and a greater demand for medical resources [1]. In the large cohort of severe COVID-19 patients during the early pandemic, 40% developed dyspnea within seven days, and 14% progressed to severe and critical illness [2]. Therefore, controlling disease progression is crucial to reduce the mortality and resolve the medical resource crisis. A hyperinflammatory response causes disease progression called a cytokine storm, resulting in multiple organ and failures [3]. Hence, immunosuppressants, particularly systemic corticosteroids, are essential, as evidenced by many studies.

A Randomized Evaluation of COVID-19 Therapy (RECOVERY) trial reported that low-dose dexamethasone (DXM) reduced time to recovery and mortality, especially in patients who needed oxygen support [4]. Subsequently, several studies were conducted using high-dose corticosteroids in moderate to critical cases and demonstrated additional benefits such as reduced systemic inflammation and ventilator days. Based on the COVID-19 dexamethasone (CoDEX) randomized clinical trial(RCT) [5], the regimen of high-dose DXM (20 mg/day) for five days, then 10 mg/day for five more days for moderate COVID-19 pneumonia (90-94% oxygen saturation on room air) was implemented as the guideline in our institute. In our practice, we found that some cases still deteriorated from hypoxemia to respiratory failure within a few days, and the laboratory markers indicated that inflammation was uncontrolled. The increased severity can be attributed to the rapid onset of organizing pneumonia caused by SARS-CoV-2. This condition has been observed in autopsies as early as the first week of infection. It typically necessitates treatment with high doses of corticosteroids, sometimes administered as initial “pulse” doses followed by extended therapy [6].

Some studies showed that high-dose methylprednisolone (MP) three to 5 days had provided better clinical improvement, decreased inflammatory markers, and had a lower proportion of patients who progressed to severe acute respiratory distress syndrome (ARDS) when compared with DXM [7, 8]. In a cohort study, Pinzón et al. compared high-dose MP for three days and oral prednisolone for 14 days against another regimen. The findings suggest that high-dose MP of 250 to 500 mg daily for three days effectively manages severe cases of COVID-19 pneumonia [7]. Corticosteroids can influence the inflammatory process at the genomic level or through faster-acting non-genomic pathways. The latter requires high doses of corticosteroids, and the response is dose-dependent. In vitro studies indicate that MP elicits a more robust response than DXM [9]. Mechanistically, MP exhibits a greater lung tissue-to-plasma ratio and is more efficient at penetrating tissue than other forms in experimental animals than dexamethasone, demonstrating higher potency in lung injury [10]. Furthermore, high-dose MP therapy did not increase adverse events in severe and critical cases, especially those concerning bacterial infection.

In moderate COVID-19 pneumonia as defined by the World Health Organization (WHO) [11], no RCT has been conducted to compare high-dose MP therapy with high-dose DXM. We hypothesized that the initial treatment with MP 250 mg daily compared with high-dose DXM based on the previous study of the CoDEX randomized clinical trial [5] for moderate COVID-19 pneumonia would improve clinical outcomes in moderate COVID-19 pneumonia patients.

Our objective was to compare the effectiveness of a high-dose MP versus a high-dose DXM in hospitalized moderate COVID-19 pneumonia, regarding the WHO clinical progression scales, mortality, and the length of hospitalization.

## Methods

### Study design and participants

This study was conducted as an open-label RCT. The trial involved the participation of two medical centers, namely Ramathibodi Hospital and Chakri Naruebodindra Medical Institute. This study was approved by the Human Research Ethics Committee, Faculty of Medicine, Ramathibodi Hospital, Mahidol University on October 11, 2021 (code COA.MURA2021/855) and registered at Thai Clinical Trials Registry on October 17, 2021, with identifier TCTR20211017001 before the first participant was enrolled. Our study adhered to CONSORT guidelines.

The study population included patients who hospitalized with moderate COVID-19 pneumonia within 48 h. To diagnose COVID-19 pneumonia, the following conditions were met: (1) identification of SARS-CoV-2 using reverse transcription polymerase chain reaction or antigen test kit in nasal swabs and sputum samples, and (2) abnormal chest X-ray (CXR) compatible with pneumonia (mostly bilateral, interstitial opacities and peripheral or subpleural ground glass opacities, predominant in mid to lower lung zones). Moderate COVID-19 pneumonia was defined as a case of COVID-19 pneumonia with a resting oxygen saturation of 90-94% [11].

### Inclusion and exclusion criteria

Study participants were required to meet the inclusion criteria: (1) aged over 18 years old, (2) hospitalized within 48 h of symptoms, (3) confirmed the diagnosis with moderated COVID-19 pneumonia as described above, and (4) provided informed consent. Individuals were excluded from the study if they met the exclusion criteria: (1) use of non-invasive or invasive mechanical ventilation (MV), (2) high risk for corticosteroids administration including other concomitant infection (bacteria, mycobacteria or fungi) and poor glycemic control, (3) Immunocompromised status including receiving immunosuppressive agents or chemotherapy, end-stage liver disease, end-stage renal disease without renal replacement therapy and Human Immunodeficiency Virus (HIV) infection with CD4 cell count less than 200 cell/mm^2^, (4) pregnant women, (5) history of active psychiatric problems, (6) current systemic corticosteroids use more than 20 mg per day of prednisolone or equivalent dose, (7) having other coexisting causes of hypoxemia, and (8) denied to participate the study.

### Randomization

Participants in the study were randomly assigned to the experimental and control groups in a ratio of 1:1. A computer-generated random list was prepared using permuted balanced blocks of four in a random sequence. A computer-based randomization system ensured that treatment assignments were concealed until a patient was enrolled in the study.

### Study protocol and outcomes

**Fig. 1:**
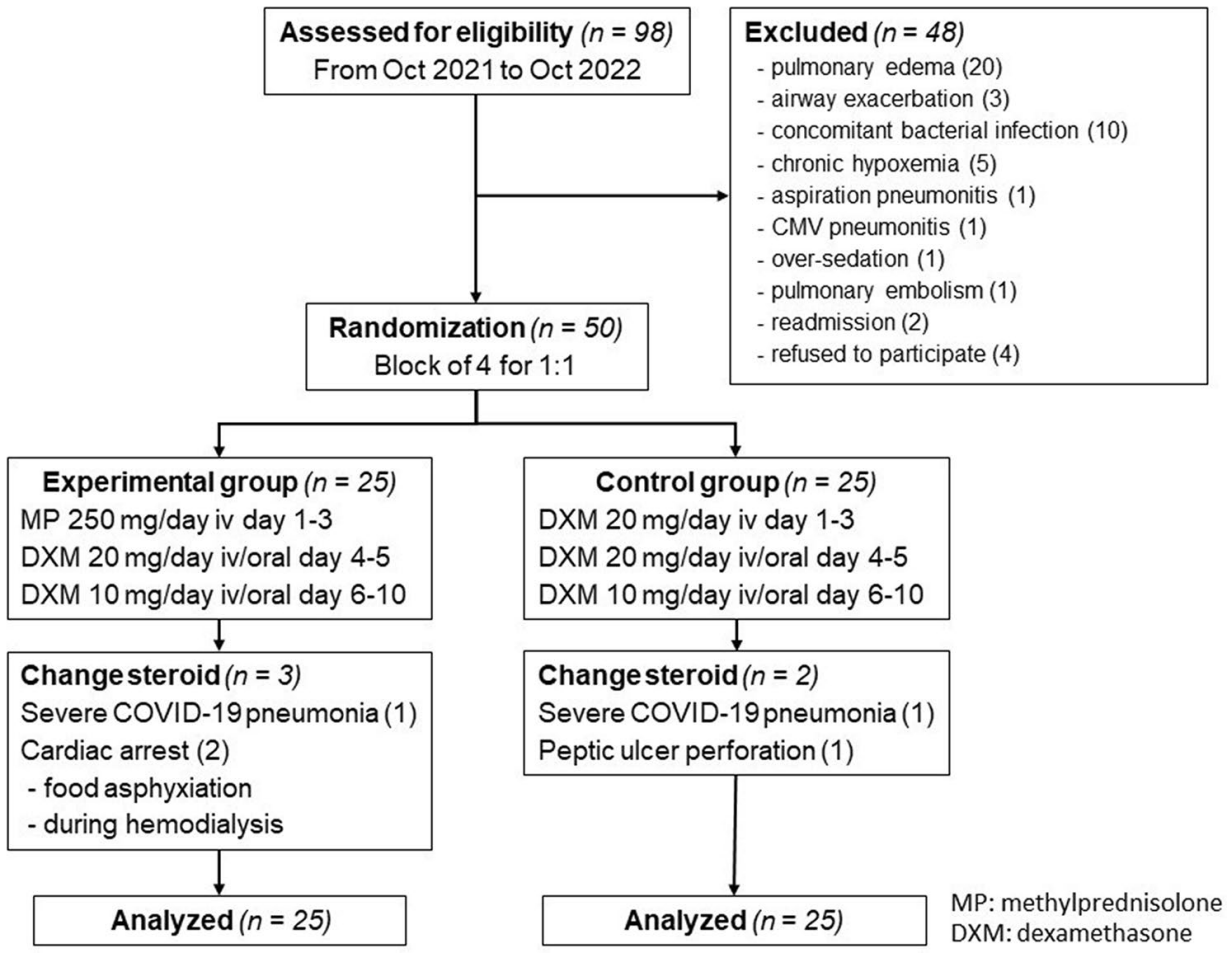
The Consolidated Standards of Reporting trials flow diagram of the study

During the first three days, participants in the experimental group received intravenous 250 mg of MP daily, while the control group received 20 mg of DXM daily. Subsequently, all participants were given oral or intravenous 20 mg of DXM daily on days 4 and 5, followed by 10 mg of DXM daily on days 6 to 10, as shown in Fig. 1. The participants in both groups received the same standard care for COVID-19 according to the practical protocol developed by Ramathibodi Hospital and Chakri Naruebodindra Medical Institute, except for corticosteroids therapy.

Clinical and demographic data, COVID-19 vaccination, blood samples, and CXR were obtained before enrollment in the study. All participants were followed up daily for clinical assessment and data collection until discharge or at least 28 days in the hospital. On day 5 and 10, participants were assessed for their clinical status, oxygenation, CXR, and blood samples, especially inflammatory markers namely the complete blood count, C-reactive protein (CRP), lactate dehydrogenase (LDH), D-dimer, and sequential organ failure assessment (SOFA) score. We utilized the WHO clinical progression scales to compare the two groups’ clinical status. These scales are a 10-point system that ranges from 0, representing no clinical or virological signs of infection (i.e., uninfected), to 10, indicating death [12]. More details regarding the WHO clinical progression scales are shown in the supplementary file. We obtained a license to use this scale from the WHO permission team. The investigators (J.L. or T.T.) assessed each patient’s WHO Clinical Progression Scales.

A primary outcome was to compare the WHO clinical progression scales on day 5 between both groups. Secondary outcomes included the difference in the WHO clinical progression scales on day 10, oxygenation improvement, transfer to the ICU, respiratory failure, complications, length of hospitalization, mortality rate, inflammatory markers, and SOFA score between the two groups.

For safety concerns, participants were terminated from the corticosteroids regimen of the assigned group if they met the criteria for termination: (1) developing severe COVID-19 pneumonia that required a higher dose than the assigned regimen (resting oxygen saturation less than 90% at room air or the need for a non-invasive or invasive MV), (2) having a contraindication for high-dose corticosteroids which was a hyperglycemic crisis and suspicion of superimposed bacterial or opportunistic infection, and (3) progression of COVID-19 pneumonia (worsening hypoxemia, radiological progression, and an increase of inflammatory markers) after the first three days, and the primary physician refused to reduce the corticosteroids dose per protocol.

All participants were followed up in the outpatient department or telemedicine care for 90 days to assess clinical status at days 14 and 28 and long-term complications at 90 days.

### Sample size calculation

Due to no previous study comparing the two corticosteroid regimens at this time, the target sample size was based on the most relevant clinical trial. According to the RCT by Ranjbar et al. [8], in comparison between the first group that received methylprednisolone and the second group receiving dexamethasone, the WHO Clinical Progression Scale on day 5 after treatment was 4.02 ± 1.64 and 5.21 ± 1.73, respectively. A target sample size of 60 participants was calculated in a 1:1 ratio, with a 95% confidence interval (CI) (first type alpha error) and 80% power. The sample size was calculated using the n4Studies program [13].

### Statistical analysis

Continuous variables were described as mean ± standard deviation or median (interquartile range, IQR), and categorical variables data were described by number (%). Statistical differences were evaluated using student *t*-tests or Mann-Whitney *U*-tests, as appropriate, for continuous variables and Pearson Chi-square tests or Fisher’s exact tests for categorical variables. The difference in the WHO clinical progression scales at days 5 and 10 was evaluated using multilevel mixed-effects linear regression and is described by a 95% confidence interval and a *p*-value. All analysis was performed using STATA version 17 and *p* < 0.05 was considered statistically significant.

## Results

### Patients

There were 98 eligible patients enrolled between October 18, 2021, and October 31, 2022, from the two centers, 42 patients were excluded due to other causes of hypoxemia as the reasons for admission, four patients refused to participate the study, and two patients were experienced secondary COVID-19 diagnoses and readmitted. We stopped the recruitment at September 2022 and had a sample size lower than expected due to a subsided outbreak during the study period. Of fifty patients who underwent randomization, 25 were assigned to the experimental group and 25 to the control group. Three patients in the experimental group and two in the control group had changed the corticosteroids regimen from protocol; two developed severe COVID-19 pneumonia, two suffered cardiac arrests, and one had a peptic ulcer perforation. Overalls were follow-up until 90 days and analyzed as intention-to-treat analysis. Based on the per-protocol analysis, the results for the outcomes did not differ from the intention-to-treat analysis. The flow diagram of the study is summarized in Fig. 1.

Of all patients, the mean age was 76.0 ± 13.3 years old, and 24 (48%) were female. The proportion of patients enrolled from each center was similar between the two groups. The median date of admission and date of illness at the day of enrollment were 2 (IQR, 1, 3) and 3 (IQR, 2, 5) days, respectively. All patients were categorized on WHO ordinal scale at 5 (hospitalized; oxygen by mask or nasal cannula). Baseline characteristics of the underlying diseases, COVID-19 vaccine status, and severity are summarized in Table 1.

**Table 1:** Baseline characteristics and severity of COVID-19 in the MP group and DXM group

Characteristics	MP group(*n* = 25)	DXM group (*n* = 25)	*p*-value
**Age** (years); mean ± S.D.	75.4 ± 12.7	76.5 ± 14.1	0.770
**Sex** (female)	11 (44%)	13 (52%)	0.571
**BMI** (kg/M^2^); mean ± S.D.	25.9 ± 6.5	23.0 ± 6.0	0.106
Obesity (BMI ≥ 30)	5 (20%)	3 (12%)	0.702
**Hypertension**	21 (84%)	21 (84%)	1.000
**Diabetes mellitus**	14 (56%)	16 (64%)	0.564
HbA1C(mg%); mean ± S.D.	6.5 ± 1.8	6.4 ± 1.5	0.843
Poorly controlled (HbA1C ≥ 9)	2 (8%)	1 (4.2%)	1.000
**Chronic heart disease**	7 (28%)	4 (16%)	0.306
**Chronic lung disease**	4 (16%)	9 (36%)	0.107
**CKD stage 4–5 or ESRD**	4 (16%)	2 (8%)	0.667
**Old cerebrovascular accident**	3 (12%)	1 (4%)	0.609
**Status bedridden**	3 (12%)	6 (24%)	0.463
**COVID-19 vaccines** (2 weeks apart)			
No vaccine	10 (40%)	6 (24%)	
1 dose	1 (4%)	2 (8%)	
2 doses	10 (40%)	13 (52%)	
3 doses	4 (16%)	4 (16%)	0.645
Included mRNA vaccine	9 (36%)	8 (32%)	0.765
**DOI** (days); median (IQR)	3 (2–5)	3 (2–6)	0.687
**Severity of COVID-19**			
**Day 1 PaO**_**2**_**/FiO**_**2**_**ratio**; mean ± S.D.	317.6 ± 34.1	326.4 ± 35.1	0.372
**Day 1 SOFA score**; median (IQR)	2 (1–3)	1 (1–2)	0.213
**Day 1 CRP(mg/L)**; median (IQR) High CRP ≥ 50	33 (14.0-63.8) 9 (36%)	22.5 (6–63) 7 (28%)	0.734 0.544
**Day 1 ALC(cells/mm3)**; median (IQR) Low ALC < 1000	1050 (800–1050) 12 (48%)	974 (614.5-1574.7) 13 (52%)	0.831 0.777
**Day 1 LDH(IU/L)**; mean ± S.D.	275.3 ± 96.2	255.1 ± 104.8	0.480
**Day 1 D-dimer(mcg/mL) **; median (IQR)	869 (483–1489)	1218 (573–1639)	0.222

Data are presented as n (%), mean (standard deviation or S.D.) or median (interquartile range or IQR). MP: methylprednisolone; DXM: dexamethasone; BMI: body mass index; HbA1C: hemoglobin A1C; CKD: chronic kidney disease; mRNA: messenger ribonucleic acid; DOA: Date of admission; DOI: Date of illness; WHO: world health organization; PaO2: arterial oxygen tension; FiO2: inspiratory oxygen fraction; SOFA score: Sequential organ failure assessment score; CRP: C-reactive protein; ALC: absolute lymphocyte count; LDH: lactate dehydrogenase

There were no significant differences in coexisting conditions, the severity of hypoxemia [Partial pressure of arterial oxygen (PaO2)/Fraction of inspired oxygen(FiO2) ratio], SOFA score, and inflammatory markers between the two groups. In addition, no significant differences in receiving essential treatments between the two groups namely, antiviral treatments, anticoagulants, and immunomodulators. (Table 2).

**Table 2:** Summary of treatments in the MP group and DXM group

Treatments	MP group (*n* = 25)	DXM group (*n* = 25)	*p*-value
**Antiviral**			
Favipiravir only	5 (20%)	10 (40%)	
Remdesivir only	12 (48%)	11 (44%)	
Both favipiravir and remdesivir	7 (28%)	4 (16%)	
Monupiravir only	1 (4%)	0 (0%)	0.325
**Extended steroids** (> 10 days)	3 (12%)	3 (12%)	1.000
**Anticoagulant**	12 (48%)	9 (36%)	0.390
**Immunomodulators**			
None	22 (88%)	22 (88%)	
Tocilizumab	1 (4%)	1 (4%)	
Baricitinib	2 (8%)	2 (8%)	1.000

Data are presented as n (%). MP: methylprednisolone; DXM: dexamethasone

### Primary outcome

**Fig. 2:**
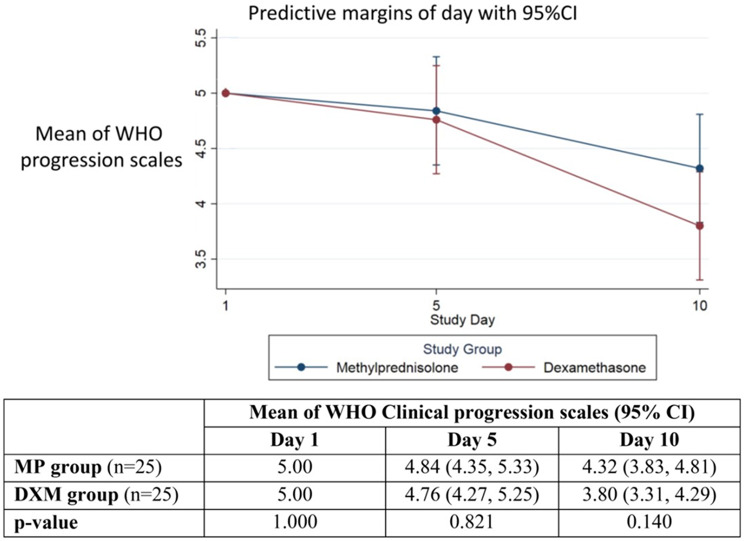
Diagram of the mean (95% confidence interval) of WHO clinical progression scales at day 1, day 5, and day 10 in the MP group and DXM group and *p*-value by multilevel mixed-effects linear regression. Data are presented as mean (95% confidence interval or 95%CI) and p-value. MP: methylprednisolone; DXM: dexamethasone

The mean of WHO clinical progression scale at day 5 was 4.84 (95% CI, 4.35–5.33) versus 4.76 (95% CI, 4.27–5.25) in the MP group and DXM group, respectively (*p* = 0.821). No significant difference in the primary outcome was observed between the two groups (Fig. 2).

### Secondary outcomes

The mean WHO clinical progression scores on day 10 were lower in the group receiving DXM than in the MP group. The scores were 3.80 (95% CI, 3.31–4.29) for the DXM group and 4.32 (95% CI, 3.83–4.81) for the MP group, but this difference was not statistically significant (*p* = 0.140) (Fig. 2). Although all secondary outcomes, including clinical improvement, oxygenation improvement, and inflammatory parameters, did not show a significant difference, the percentage of patients with a lower PaO2/FiO2 ratio on the 5th and 10th days of treatment was lower in the DXM group according to Table 3, but this also did not reach statistical significance.

**Table 3:** Secondary outcomes in the MP group and DXM group

Secondary outcomes	MP group (*n* = 25)	DXM group (*n* = 25)	*p*-value
**Day 5 PaO**_**2**_**/FiO**_**2**_**ratio**; mean ± S.D.	355.6 ± 79.1	338.9 ± 82.9	0.474
Hypoxemia PaO_2_/FiO_2_ ratio > 300	75%	64%	
PaO_2_/FiO_2_ ratio > 200–300	16.7%	32%	
PaO_2_/FiO_2_ ratio 100–200	8.3%	4%	0.478
**Day 10 PaO**_**2**_**/FiO**_**2**_**ratio**; mean ± S.D.	369.0 ± 77.7	388.6 ± 72.3	0.370
Hypoxemia PaO_2_/FiO_2_ ratio > 300	75%	83.3%	
PaO_2_/FiO_2_ ratio > 200–300	25%	16.7%	0.477
**Day 5 CRP(mg/L)**; median (IQR)	7.2 (5.4–17.8)	7.8 (5.5–24.3)	0.667
Normalized CRP < 10 mg/dl	13 (54.2%)	13 (52%)	0.879
**Day 10 CRP**; median (IQR)	6 (2.9–13.7)	6 (3.4–18.0)	0.844
Normalized CRP < 10 mg/dl	16 (66.7%)	14 (58.3%)	0.551
**Day 5 LDH(IU/L)**; mean ± S.D.	264 ± 87.3	240.4 ± 95.7	0.368
**Day 10 LDH**; mean ± S.D.	254.9 ± 91.8	237.2 ± 82.0	0.484
**Day 5 SOFA score**; median (IQR)	1 (1-2.5)	1 (1–2)	0.883
**Day 10 SOFA score**; median (IQR)	1 (0.5-2)	0 (0–2)	0.103
**Day 5 D-dimer(mcg/mL)**; median (IQR)	846.5 (470–2492)	1021 (474–1996)	0.976
**Day 10 D-dimer**; median (IQR)	1043 (594.5–1712)	1189 (576.5-3880.5)	0.446
**Being alive with SaO**_**2**_ **≥ 96% room air**			
Day 14	16 (64%)	18 (72%)	0.544
Day 28	21 (84%)	21 (84%)	1.000
**Being alive without mechanical ventilation**			
Day 14	23 (92%)	23 (92%)	1.000
Day 28	22 (88%)	22 (88%)	1.000
**Being alive without hospitalization**			
Day 14	16 (64%)	18 (72%)	0.544
Day 28	21 (84%)	20 (80%)	1.000
**Transfer to intensive care unit**	3 (12%)	3 (12%)	1.000
**Acute respiratory failure**	2 (8%)	2 (8%)	1.000
**Length of hospitalization** (days)			
Excluded hospital death; median (IQR)	13 (10–18)	11 (9–13)	0.227
**Hospital mortality**	2 (8%)	2 (8%)	1.000
**28-day mortality**	3 (12%)	2 (8%)	1.000
**90-day mortality**	4 (16%)	3 (12%)	1.000

Data are presented as n (%), mean (standard deviation or S.D.) or median (interquartile range or IQR). MP: methylprednisolone; DXM: dexamethasone; PaO2: arterial oxygen tension; FiO2: inspiratory oxygen fraction; CRP: C-reactive protein; LDH: lactate dehydrogenase; SOFA score: Sequential organ failure assessment score; SaO_2_ : Oxygen saturation

The majority of patients in both groups had the PaO2/FiO2 > 300 at day 5, 18 (75%) in MP group and 16 (64%) in DXM group; and at day 10, 18 (75%) in MP group and 20 (83.3%) in DXM group. CRP levels, as well as LDH, D-dimer and SOFA scores, were not significantly different between the two groups at day 5 and day 10 (Table 3).

Clinical improvements were similar between the two groups at 14-day and 28-day follow-ups. In both groups, there were the same rates of ICU transfer (12%), the development of respiratory failure (8%), and in-hospital mortality (8%). In addition, there were no significant differences between the MP group and the DXM group in the length of hospitalization (13 days versus 11 days, *p* = 0.227), 28-day mortality (12% versus 8%, *p* = 1.000), and 90-day mortality (16% versus 12%, *p* = 1.000).

Table 4 shows no statistically significant difference of complications occurred during hospitalization and until 90 days follow-up. There was a trend towards fewer patients with septic shock in the MP group compared to the DXM group (0% versus 16%, *p* = 0.110). Regarding the causes of septic shock in the DXM group, two patients had septic shock from hospital-acquired pneumonia, one from urinary tract infection, and one from peptic ulcer perforation. One patient in the DXM group was diagnosed with a hyperglycemia crisis 22 days after admission.

**Table 4:** Summary of complications in the MP group and DXM group

Complications	MP group (*n* = 25)	DXM group (*n* = 25)	*p*-value
Pulmonary complications			
HAP or VAP	0 (0%)	1 (4%)	1.000
AFOP	3 (12%)	2 (8%)	1.000
Lobar atelectasis	1 (4%)	2 (8%)	1.000
Tracheostomy	0 (0%)	1 (4%)	1.000
**Systemic complications**			
Acute kidney injury	4 (16%)	7 (28%)	0.306
Septic shock	0 (0%)	4 (16%)	0.110
Hyperglycemic crisis	0 (0%)	1 (4%)	1.000
**90-day opportunistic infections**	0 (0%)	0 (0%)	-
**90-day long term oxygen therapy**	0 (0%)	0 (0%)	-

Data are presented as n (%). MP: methylprednisolone; DXM: dexamethasone; HAP: hospital acquired pneumonia; VAP: ventilator associated pneumonia; AFOP: acute fibrinous organizing pneumonia

## Discussion

In this open-label RCT, we compared high-dose MP with high-dose DXM for the first three days, followed by the same DXM regimen until ten days in patients with moderate COVID-19 pneumonia. The WHO progression scales on day 5 did not differ significantly between the two groups. The mean WHO clinical progression scores on day 10 were lower in the group receiving DXM than the MP group, but this difference was not statistically significant. There were no significant differences in secondary outcomes, such as clinical improvement in the hospital until 28 days after enrollment, oxygenation improvement, inflammatory markers, respiratory failure, ICU transfer, and mortality. Although the percentage of patients with a lower PaO2/FiO2 ratio on the 5th and 10th days of treatment was lower in the DXM group, according to Table 3, this also did not reach statistical significance. Also, superimposed bacterial or opportunistic infections and hyperglycemic crises are not significantly different.

Systemic corticosteroids are well-known and readily available anti-inflammatory medications that much evidence has shown to be effective in COVID-19 patients. Based on the RECOVERY trial, a medium-dose regimen (6 mg of DXM once daily) was beneficial only for COVID-19 patients who received respiratory support or had inflammation that required control [4]. Subsequently, several studies reported that a high-dose to pulse regimen was also beneficial in COVID-19 patients with severe and critical illnesses and appeared to improve outcomes more than a medium-dose regimen [5, 7, 8, 14, 15]. Based on the evidence, three main factors should be considered to predict the advantage of systemic corticosteroids: (1) degree of inflammation, (2) dose of corticosteroids, and (3) drug tissue concentration.

Firstly, COVID-19 patients who manifest severe to critical illness or meet hyperinflammatory response conditions will deteriorate as these conditions indicate that anti-inflammatory treatment is required to mitigate the inflammatory process. This is important not only in the early phase of COVID-19 pneumonia but also ARDS in the terminal phase of infection to lessen inflammation and tissue injury [16]. In the early stage of COVID-19 pneumonia, the timing of administration is determined by the benefit of inflammation control over the risk of prolonged viral shedding, which has to be discussed. Studies by Raef Fadel et al. and Pablo Monedero et al. have shown that early (within 48 h) corticosteroids use in moderate to critically ill patients results in lower mortality, shorter stays in ICU, decreased organ dysfunction, and fewer days on MV [15, 17]. Secondly, the anti-inflammatory effect of corticosteroids depends on their dose categorized by mg of prednisolone equivalent dose per day: low-dose (≤ 7.5 mg), medium-dose (> 7.5 mg and ≤ 30 mg), high-dose (> 30 mg), very high-dose (> 100 mg), and pulse therapy (≥ 250 mg) [18]. Corticosteroids have a dual action pathway: they inhibit inflammation through genomic and non-genomic pathways. The treatment regimen, which involved a prednisolone equivalent dose exceeding 100 mg, demonstrated significant non-genomic potency, contributing to rapidly suppressing the inflammation. It was proven in a triple-blind RCT by Keivan Ranjbar et al. that a very high-dose regimen was more effective than a medium-dose one (as in the RECOVERY trial) for patients with moderate COVID-19 pneumonia [8]. Additionally, it does not increase the risk of infections that most studies have reported. Thirdly, MP might have contributed to better outcomes because of its tendency to concentrate in lung tissue more than DXM [18–22]. In COVID-19, however, no clinical trial has been established to support this issue.

Regarding the results from our study, there was no difference in overall clinical outcomes between the two corticosteroids groups. The main reason explaining our results could be that our patients’ disease progression was less severe than in the previous studies. A significantly lower incidence of respiratory failure, ICU transfer, and the mortality rate was observed in our study, when compared the the previous study [5].

The population in our study includes both vaccinated and unvaccinated individuals. Over half of the patients already had vaccinations. A recent study reported that patients in the unvaccinated group were more likely to develop pneumonia, require supplemental oxygen, and have a longer time from symptom onset to hospital discharge than the vaccinated group [23]. Thus, it’s crucial to explicitly address and control the effect of prior vaccination on the progression and treatment response of COVID-19. We performed additional sensitivity analyses to compare the mean differences (M.D.) and their 95% CI, along with p-values for WHO scores on day 5 and day 10, both unadjusted and adjusted for prior vaccination status, between the two groups. Both unadjusted and adjusted M.D. of WHO scores on day 5 and day 10 are small and not statistically significant (p-values 0.79 and 0.61, respectively), suggesting no meaningful difference between the groups (Supplementary Table 1 in supplementary file).

Additionally, the clinician had gained experience and knowledge, and the omicron variant, less virulent than the delta variant, was predominant in the study period. Because of less inflammation, the power of different potencies may not be visible. In comparable studies, multicenter RCTs were conducted in moderate COVID-19 pneumonia, similar to ours. Those studies compared pulse therapy to a medium-dose regimen (6 mg of DXM), with no significant differences in the outcomes [24, 25]. Hence, a medium dose of corticosteroids may be enough for anti-inflammatory treatment in moderate COVID-19 especially patients requiring oxygen therapy without invasive MV.

Despite the overall negative results of our study, we cannot exclude any potential benefits in specific subgroups of patients with more severe illnesses and more inflammatory responses. A recent clinical cohort study by Takuhiro Moromizato et al. found that pulse MP at 500 to 1,000 mg per day was significantly associated with a lower in-hospital mortality rate among patients receiving invasive MV but not those without invasive MV [26]. Therefore, pulse therapy could be a rescue therapy when critically ill patients require invasive MV. However, this should be demonstrated through a randomized controlled trial.

One of the strengths is this trial is the homogeneity of the sample, in that all patients were categorized into group 5 of the WHO clinical progression scales and had balanced baseline characteristics regarding age and comorbidities. Moreover, most patients were strictly followed and analyzed according to protocol.

### Limitations

This study’s limitation is that it was conducted at only two centers, which may limit the generalizability of the findings to other settings and populations. The sample size was lower than expected due to a subsided outbreak during the study period. However, the sample size was slightly less than expected (50 out of 60 patients or 83%), and the results’ trend was evident. The omicron variant, less virulent than the delta variant, was predominant in the study period, which could have influenced the outcomes and might not have applied to periods dominated by more virulent variants like Delta. Recently, several variants of SARS-CoV-2, such as BA.2.86 and JN.1, have emerged, resulting in reduced disease severity, as evidenced by ICU admissions per 1000 hospitalizations decreasing since the peak in July 2021 [27]. This development prompts investigation into the necessity of high-dose steroids for this patient group.

Regarding the blinding, each patient’s WHO clinical progression scale was not blinded and assessed by the investigators in both centers (J.L. and T.T.) Nevertheless, the WHO Clinical Progression Scale depends on the requirement of oxygen therapy, mechanical ventilation, and ambulatory status. It relies on the decision of the primary physician, who was not involved in the studies.

The patients were followed up to 90 days. Considering the potential long-term effects of COVID-19 and corticosteroid use, multicenter prospective trials with extended follow-up would be valuable and provide more robust and generalizable data.

## Conclusion

In patients with moderate COVID-19 pneumonia, initial anti-inflammatory treatment with 250 mg per day of MP for three days was not more effective than the high-dose DXM regimen.

## Electronic supplementary material

Below is the link to the electronic supplementary material.

Supplementary Material 1

## Data Availability

The data can be requested from the corresponding author.

## Abbreviations

DXM: Dexamethasone
MP: Methylprednisolone
WHO: World Health Organization
RCT: Randomized controlled trial
CXR: Chest X-ray
MV: Mechanical ventilation
HIV: Human Immunodeficiency Virus
CRP: C-reactive protein
LDH: Lactate dehydrogenase
SOFA: Sequential organ failure assessment
IQR: Interquartile range
PaO2: Partial pressure of arterial oxygen
FiO2: Fraction of inspired oxygen
CI: Confidence interval

## References

[1] Gandhi RT, Lynch JB, Del Rio C. Mild or moderate Covid-19. N Engl J Med. 2020;383(18):1757–66. 32329974 10.1056/NEJMcp2009249

[2] Berlin DA, Gulick RM, Martinez FJ. Severe Covid-19. N Engl J Med. 2020;383(25):2451–60. 32412710 10.1056/NEJMcp2009575

[3] Fajgenbaum DC, June CH. Cytokine storm. N Engl J Med. 2020;383(23):2255–73. 33264547 10.1056/NEJMra2026131 PMC7727315

[4] Horby P, Lim WS, Emberson JR, Mafham M, Bell JL, Linsell L, et al. Dexamethasone in hospitalized patients with Covid-19. N Engl J Med. 2021;384(8):693–704. 32678530 10.1056/NEJMoa2021436 PMC7383595

[5] Tomazini BM, Maia IS, Cavalcanti AB, Berwanger O, Rosa RG, Veiga VC, et al. Effect of dexamethasone on days alive and Ventilator-Free in patients with moderate or severe Acute Respiratory Distress Syndrome and COVID-19: the CoDEX Randomized Clinical Trial. JAMA. 2020;324(13):1307–16. 32876695 10.1001/jama.2020.17021 PMC7489411

[6] Kory P, Kanne JP. SARS-CoV-2 organising pneumonia: ‘Has there been a widespread failure to identify and treat this prevalent condition in COVID-19?‘. BMJ open Respiratory Res. 2020;7(1). 10.1136/bmjresp-2020-000724 PMC7509945 32963028

[7] Pinzón MA, Ortiz S, Holguín H, Betancur JF, Cardona Arango D, Laniado H, et al. Dexamethasone vs methylprednisolone high dose for Covid-19 pneumonia. PLoS ONE. 2021;16(5):e0252057. 34033648 10.1371/journal.pone.0252057 PMC8148307

[8] Ranjbar K, Moghadami M, Mirahmadizadeh A, Fallahi MJ, Khaloo V, Shahriarirad R, et al. Methylprednisolone or dexamethasone, which one is superior corticosteroid in the treatment of hospitalized COVID-19 patients: a triple-blinded randomized controlled trial. BMC Infect Dis. 2021;21(1):337. 33838657 10.1186/s12879-021-06045-3 PMC8035859

[9] Ayyar VS, Jusko WJ. Transitioning from Basic toward systems Pharmacodynamic models: lessons from corticosteroids. Pharmacol Rev. 2020;72(2):414–38. 32123034 10.1124/pr.119.018101 PMC7058984

[10] Ayyar VS, Song D, DuBois DC, Almon RR, Jusko WJ. Modeling corticosteroid pharmacokinetics and pharmacodynamics, part I: determination and prediction of Dexamethasone and Methylprednisolone tissue binding in the rat. J Pharmacol Exp Ther. 2019;370(2):318–26. 31197020 10.1124/jpet.119.257519 PMC6658919

[11] Organization WHOJWH. Clinical management of COVID-19: living guidance. 2021.

[12] A minimal common outcome measure set for COVID-19 clinical research. Lancet Infect Dis. 2020;20(8):e192–7. 32539990 10.1016/S1473-3099(20)30483-7 PMC7292605

[13] Ngamjarus C. n4Studies: sample size calculation for an epidemiological study on a smart device. Siriraj Med J. 2016;68(3):160–70.

[14] Edalatifard M, Akhtari M, Salehi M, Naderi Z, Jamshidi A, Mostafaei S et al. Intravenous methylprednisolone pulse as a treatment for hospitalised severe COVID-19 patients: results from a randomised controlled clinical trial. Eur Respir J. 2020;56(6). 10.1183/13993003.02808-2020 PMC7758541 32943404

[15] Fadel R, Morrison AR, Vahia A, Smith ZR, Chaudhry Z, Bhargava P, et al. Early short-course corticosteroids in hospitalized patients with COVID-19. Clin Infect Diseases: Official Publication Infect Dis Soc Am. 2020;71(16):2114–20. 10.1093/cid/ciaa601 PMC7314133 32427279

[16] Mehta J, Rolta R, Mehta BB, Kaushik N, Choi EH, Kaushik NK. Role of Dexamethasone and Methylprednisolone corticosteroids in Coronavirus Disease 2019 hospitalized patients: a review. Front Microbiol. 2022;13:813358. 35242118 10.3389/fmicb.2022.813358 PMC8886296

[17] Monedero P, Gea A, Castro P, Candela-Toha AM, Hernández-Sanz ML, Arruti E, et al. Early corticosteroids are associated with lower mortality in critically ill patients with COVID-19: a cohort study. Crit Care (London England). 2021;25(1):2. 10.1186/s13054-020-03422-3 PMC7780210 33397463

[18] Buttgereit F, da Silva JA, Boers M, Burmester GR, Cutolo M, Jacobs J, et al. Standardised nomenclature for glucocorticoid dosages and glucocorticoid treatment regimens: current questions and tentative answers in rheumatology. Ann Rheum Dis. 2002;61(8):718–22. 12117678 10.1136/ard.61.8.718 PMC1754188

[19] Bajaj D, Gupta M, Manek G, Manek G, Hu K, Boregowda UJIJCCEM. Dexamethasone versus methylprednisolone in hospitalized COVID-19 patients: a systematic review and meta-analysis. Int. J. Crit. Care Emerg. Med. 2021;7:128.

[20] Braude AC, Rebuck AS. Prednisone and methylprednisolone disposition in the lung. Lancet (London England). 1983;2(8357):995–7. 6138595 10.1016/s0140-6736(83)90981-9

[21] Hirano T, Homma M, Oka K, Tsushima H, Niitsuma T, Hayashi T. Individual variations in lymphocyte-responses to glucocorticoids in patients with bronchial asthma: comparison of potencies for five glucocorticoids. Immunopharmacology. 1998;40(1):57–66. 9776479 10.1016/s0162-3109(98)00025-3

[22] Vichyanond P, Irvin CG, Larsen GL, Szefler SJ, Hill MR. Penetration of corticosteroids into the lung: evidence for a difference between methylprednisolone and prednisolone. J Allergy Clin Immunol. 1989;84(6 Pt 1):867–73. 2600321 10.1016/0091-6749(89)90381-3

[23] Seo WJ, Kang J, Kang HK, Park SH, Koo HK, Park HK, et al. Impact of prior vaccination on clinical outcomes of patients with COVID-19. Emerg Microbes Infections. 2022;11(1):1316–24. 10.1080/22221751.2022.2069516 PMC9132471 35465831

[24] Corral-Gudino L, Cusacovich I, Martín-González JI, Muela-Molinero A, Abadía-Otero J, González-Fuentes R, et al. Effect of intravenous pulses of methylprednisolone 250 mg versus dexamethasone 6 mg in hospitalised adults with severe COVID-19 pneumonia: an open-label randomised trial. Eur J Clin Invest. 2023;53(1):e13881. 36169086 10.1111/eci.13881 PMC9538428

[25] Salvarani C, Massari M, Costantini M, Merlo DF, Mariani GL, Viale P et al. Intravenous methylprednisolone pulses in hospitalised patients with severe COVID-19 pneumonia: a double-blind, randomised, placebo-controlled trial. Eur Respir J. 2022;60(4). 10.1183/13993003.00025-2022 PMC8971731 35361632

[26] Moromizato T, Sakaniwa R, Tokuda Y, Taniguchi K, Shibuya K. Intravenous methylprednisolone pulse therapy and the risk of in-hospital mortality among acute COVID-19 patients: Nationwide clinical cohort study. Crit Care (London England). 2023;27(1):53. 10.1186/s13054-023-04337-5 PMC9906603 36755340

[27] World Health Organization. COVID-19 situation reports. Geneva: World Health Organization; [date accessed]. https://iris.who.int/handle/10665/378047

