# Supplementary material for: A comparative study between methylprednisolone versus dexamethasone as an initial anti-inflammatory treatment of moderate COVID-19 pneumonia: an open-label randomized controlled trial
Source: BMC Pulm Med. 2024 Nov 11;24:562. doi: 10.1186/s12890-024-03364-4 (PMC11555798; doi:10.1186/s12890-024-03364-4)
Supplement: Supplementary file 1 — Supplementary Material 1 [file 12890_2024_3364_MOESM1_ESM.docx]

**WHO Clinical Progression Scales**

The WHO Clinical Progression Scale for COVID-19 is a standardized tool developed to categorize the severity and progression of COVID-19 in patients. It ranges from 0 to 10, where each score indicates a specific level of disease severity and clinical status:

**0** - Uninfected: no clinical or virological evidence of infection.

**1** - Ambulatory: no limitation of activities.

**2** - Ambulatory: limitation of activities.

**3** - Ambulatory: hospitalization is not required.

**4** - Hospitalized: no oxygen therapy is required.

**5** - Hospitalized: oxygen by mask or nasal prongs.

**6** - Hospitalized: oxygen by non-invasive ventilation or high flow.

**7** - Intubation and mechanical ventilation.

**8** - Mechanical ventilation and additional organ support (e.g., vasopressors, renal replacement therapy).

**9** - Mechanical ventilation, vasopressors, dialysis, or extracorporeal membrane oxygenation (ECMO).

**10** - Death.

**Supplementary Table 1**: Sensitivity analyses to compare the mean differences and their 95% confidence interval, along with p-values for WHO clinical progression scores on day 5 and day 10, unadjusted and adjusted for prior vaccination status, between the two groups**.**

| **WHO clinical progression scores** | **MD** | **95% CI** | ***p*-value** | **Adjusted MD** | **95% CI** | ***p*-value** |
| --- | --- | --- | --- | --- | --- | --- |
| **Day 5** | 0.08 | -0.51 - 0.67 | 0.79 | 0.15 | -0.45 - 0.75 | 0.61 |
| **Day 10** | 0.52 | -0.59 - 1.6 | 0.35 | 0.50 | -0.63 - 1.63 | 0.38 |

MD = Methylprednisolone group – Dexamethasone group

MD: mean difference; CI: confidence interval
